# Why Has the Number of Scientific Retractions Increased?

**DOI:** 10.1371/journal.pone.0068397

**Published:** 2013-07-08

**Authors:** R. Grant Steen, Arturo Casadevall, Ferric C. Fang

**Affiliations:** 1 MediCC!, Medical Communications Consultants, LLC Chapel Hill, North Carolina, United States of America; 2 Albert Einstein College of Medicine of Yeshiva University Bronx, New York, United States of America; 3 University of Washington School of Medicine Seattle, Washington, United States of America; Consejo Superior de Investigaciones Cientifics, Spain

## Abstract

**Background:**

The number of retracted scientific publications has risen sharply, but it is unclear whether this reflects an increase in publication of flawed articles or an increase in the rate at which flawed articles are withdrawn.

**Methods and Findings:**

We examined the interval between publication and retraction for 2,047 retracted articles indexed in PubMed. Time-to-retraction (from publication of article to publication of retraction) averaged 32.91 months. Among 714 retracted articles published in or before 2002, retraction required 49.82 months; among 1,333 retracted articles published after 2002, retraction required 23.82 months (p<0.0001). This suggests that journals are retracting papers more quickly than in the past, although recent articles requiring retraction may not have been recognized yet. To test the hypothesis that time-to-retraction is shorter for articles that receive careful scrutiny, time-to-retraction was correlated with journal impact factor (IF). Time-to-retraction was significantly shorter for high-IF journals, but only ∼1% of the variance in time-to-retraction was explained by increased scrutiny. The first article retracted for plagiarism was published in 1979 and the first for duplicate publication in 1990, showing that articles are now retracted for reasons not cited in the past. The proportional impact of authors with multiple retractions was greater in 1972–1992 than in the current era (p<0.001). From 1972–1992, 46.0% of retracted papers were written by authors with a single retraction; from 1993 to 2012, 63.1% of retracted papers were written by single-retraction authors (p<0.001).

**Conclusions:**

The increase in retracted articles appears to reflect changes in the behavior of both authors and institutions. Lower barriers to publication of flawed articles are seen in the increase in number and proportion of retractions by authors with a single retraction. Lower barriers to retraction are apparent in an increase in retraction for “new” offenses such as plagiarism and a decrease in the time-to-retraction of flawed work.

## Introduction

Science is said to be self-correcting, in that the literature can purge itself of articles deemed to be seriously flawed [1], [2]. One of the major mechanisms of self-correction is retraction of flawed work [3], [4], and the rate of retraction of scientific articles has risen sharply in recent years [5]–[7]. A substantial fraction of all retractions are due to research misconduct [8], [9] and there has been an estimated 10-fold increase in retractions for scientific fraud (*e.g*., data fabrication or falsification) since 1975 [8]. Furthermore, fraud was found to be involved in 94% of the 228 cases of misconduct identified by the U.S. Office of Research Integrity from 1994–2012 [10].

An explanation for the apparent increase in the rate of fraud is not immediately obvious. If the literature truly does self-correct, then research fraud should ultimately be futile [4]. Yet there is reasonable evidence that scientific misconduct is both common and under-reported [11]. An anonymous survey of 2,000 psychologists estimated that the prevalence of data falsification was 9%, although only 1.7% of respondents actually admitted having falsified data [12]. Among 3,247 scientists surveyed anonymously in the United States, 0.3% admitted to falsifying data and 1.4% admitted to plagiarism [13]. A survey of 125 corresponding authors, all of whom had published an article in a major medical journal, found that 5 respondents (4%) had discovered fraudulent data in their own article after publication [14]. A survey of 2,212 scientists revealed 201 instances of likely research misconduct over a 3-year period, for an incidence rate of roughly 3% per year [15]. Among 163 professional biostatisticians, 31% had been involved in a fraudulent project and 13% had been requested to “support fraud” during their research career [16]. A meta-analysis of 11,647 scientists reported in 21 separate studies concluded that 2% of scientists had committed research fraud at least once in their career [17]. If these numbers are credible, then there may be many fraudulent papers that have not been retracted [4].

Therefore, it is not clear whether the increase in retractions is a result of an increase in the rate of publication of flawed articles or an increase in the rate at which flawed articles are recognized and withdrawn [5]. The goal of this study is to gain a deeper understanding of the increase in retracted scientific publications by analyzing trends in the time interval from publication to retraction. We show that, while retractions have increased strikingly in recent years, there is reason to expect that this reflects changes in institutional behavior as well as changes in the behavior of authors.

## Methods

The PubMed database of the National Center for Biotechnology Information was searched on 3 May 2012, using the limits of “retracted publication, English language.” A total of 2,047 articles were identified, all of which were exported from PubMed and entered in an Excel database [8]. Each article was classified according to the cause of retraction, using published retraction notices, proceedings from the Office of Research Integrity (ORI), *Retraction Watch* (http://retractionwatch.wordpress.com), and other sources (*e.g*., the *New York Times*). Retractions were classified as resulting from fraud (*e.g*., data fabrication or falsification), suspected fraud, scientific error, plagiarism, duplicate publication, other cause (*e.g*., publisher error, authorship disputes, copyright infringement), or unknown. Fabrication is defined as the manufacture of fictional data, while falsification is defined as selective manipulation of actual data to present a misleading result [4]. Each assessment of the reason for retraction was reviewed by all authors and discrepancies were resolved by consensus. The initial analysis of these data is summarized in a separate manuscript, which concluded that the majority of retractions were due to misconduct [8].

The present study focused on the time required to retract a flawed article, in order to test several *a priori* hypotheses. An apparent increase in recent retractions might result: (1) if the time to retract has increased in recent years, so that editors are reaching further back in time to retract (*e.g*., if the introduction of plagiarism-detection software has lead to the detection of long-published articles that need to be retracted for plagiarism); (2) if peer scrutiny has increased, so that flawed work is detected more quickly; or (3) if there are reduced barriers to retraction, such that retraction occurs more swiftly (or for different reasons) now than in the past. The time required to retract an article was calculated as the number of months from when a hard-copy version of the article was published in a journal (*i.e*., as opposed to an online electronic version) to when the retraction notice was published.

To determine the impact of authors with multiple retractions, each first author was compared to other first and senior (last) authors. In cases where names were highly similar, research topics and institutional affiliations were used to determine whether the same author was involved. For example, there were 3 retracted papers written by an author named “Z. Shen.” One paper [18] was about defective transcription of Foxp3 in patients with psoriasis and was submitted from the Third Military Medical University in Chongqing, China. Two papers [19], [20] were about nanoembossed ferroelectric nanowires and came from Fudan University in Shanghai. It was judged that the same “Z. Shen” wrote the latter two papers, but a different “Z. Shen” wrote the former paper.

In the course of identifying whether each first author had also written other retracted papers, it was often possible to identify networks of collaborating authors. In the case of “Z. Shen” above, we noted that the senior author of the psoriasis paper was “Y. Liu,” whereas the senior author of the nanowire papers was “R. Liu.” Our approach therefore identified “R. Liu” as a senior author who had collaborated on at least 2 retractions. As the entire list of 2,047 retracted first authors was reviewed, networks of collaborating authors were identified. The number of retracted articles by each first author was tallied to determine the number of first authors with only 1 retraction, first authors with 2 to 5 retractions, and first authors with more than 5 retractions.

To determine the sensitivity of our analysis to authors with multiple retractions, we sorted first authors by name, to determine how many retractions were associated with each first author. We then compared first authors with a single retraction to first authors with multiple retractions.

All statistical tests and data plots used the capabilities native to Excel (Microsoft Office). Correlation coefficients (Table 1) were tested for significance using the R statistic, which has a t-distribution. The mathematical model used to predict the number of articles likely to be retracted in the future was derived *de novo* from consideration of the cumulative probability of retraction.

**Table 1:** Correlations among journal impact factor (IF) and time-to-retraction expressed in months for different infractions.

		Journal IF		Months to retract		Correlation r		
	Sample n	Mean	SD	Mean	SD	IF×Months	R =	P<
**Misconduct+Poss. misconduct**	889	8.71	10.08	43.03	37.40	−0.079	−2.39	0.01
**Misconduct**	697	9.10	10.24	46.78	38.38	−0.120	−3.19	0.01
**Possible misconduct**	192	7.31	9.38	29.41	29.97	0.030	0.41	NS
**Plagiarism**	200	2.63	2.42	26.04	32.55	−0.134	−1.90	0.05
**Error**	437	10.98	11.61	26.03	27.95	0.029	0.60	NS
**Duplicate publication**	290	3.91	6.33	26.61	29.63	−0.027	0.46	NS
**All retractions**	2047	7.30	9.54	32.91	34.24	−0.027	1.22	NS

This table includes all retracted articles. “Misconduct+Poss. misconduct” includes both “Misconduct” and “Possible misconduct,” which are also analyzed separately. The correlation coefficient r is tested for significance with the R statistic, which has a t-distribution. Numbers do not sum because this table does not include “other” and “unknown” infractions, and because some papers were retracted for more than one infraction.

## Results

**Figure 1:**
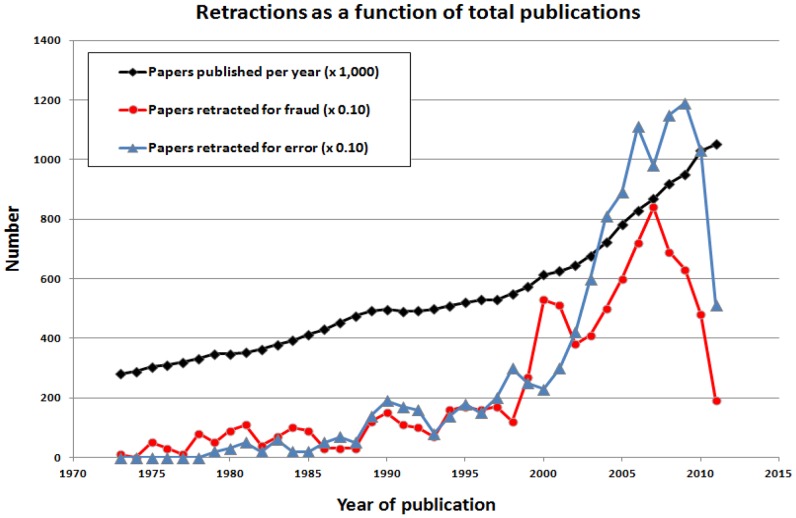
Papers published and retracted per year since 1973. Note that the multipliers are different. For the sake of simplicity, error here includes all infractions except fraud (*e.g*., scientific error, plagiarism, duplication, other). Apparent declines in recent years must be interpreted with caution as additional papers may be retracted in the future, thereby reversing this decline.

The number of articles retracted each year has risen sharply in the last decade, including both articles retracted for fraud and articles retracted for error (Fig. 1). For simplicity, error in Fig. 1 includes any type of infraction except fraud (*e.g*., scientific error, plagiarism, duplication, other). The mean time-to-retraction for a journal article is 32.91 months overall (±34.24 SD, range <1 to 304 months).

It is important to note that a single paper may be retracted for multiple reasons. A total 1,993 papers were retracted for a single infraction, 48 papers were retracted for two infractions, and 6 papers were retracted for three infractions. No papers were retracted for more than three infractions.

Several mechanisms may be contributing to the increase in retractions. One hypothesis is that the increase in retractions merely reflects an increase in total publications. To test this possibility, we determined the number of publications indexed by PubMed each year, and compared these values to the annual number of retractions for fraud and error (Fig. 1). Between 1973 and 2011, 21.2 million journal articles were published, and 890 articles were retracted for fraud, yielding a retraction rate of 1 in 23,799 articles, or approximately 0.004%. In 1973, 281,100 articles were published; in 2011, 1,051,000 articles were published, representing a nearly 4-fold increase. From 1973 to 2011 inclusive, the change in publication rate was greater (slope m = 0.051) than the change in retraction rate for fraud (m = 0.039) or error (m = 0.025). However, the rate of increase in retractions for fraud and error was greater than the rate of increase in publications between 1995 and 2005, implying a true acceleration in retractions over this time period.

An apparent (but artifactual) glut of retractions might be created if editors began to reach further back in time to retract articles. To test this hypothesis, we evaluated the average time-to-retraction for the most recent decade (2003–2012) compared to the preceding time interval from 1973 to 2002. The mean time-to-retraction for the 1,595 articles retracted in the current decade was 33.81 months (±35.63 SD), compared to 29.60 months (±28.54 SD) for the 452 articles retracted between 1973 and 2002 (two-tailed t-test, p = 0.0208). These results are consistent with the notion that journals have begun to reach back further in time to retract articles, though this trend is unlikely to account for much of the recent spike in retractions.

Widespread use of the internet has been hypothesized to increase the level of scrutiny given to published articles [7], [21], [22]. To test the hypothesis that the time-to-retraction is shorter for articles given a high level of scrutiny, the number of months to retraction was correlated with the impact factor (IF) of the journal in which an article was published (“Journal IF”), as a measure of article visibility (Table 1). We postulate that IF is a reasonable surrogate for peer scrutiny as high-IF journals are cited more widely because they are seen more widely. When all retractions were pooled, no correlation was observed between time-to-retraction and journal IF. However, the correlation between the time-to-retraction and journal IF was significant for plagiarism (p<0.05) and for all misconduct (p<0.01). The latter correlation was driven by a significant correlation with fraud (p<0.01), as this correlation was not significant for suspected fraud. Although publication in a high-IF journal did appear to shorten the time-to-retraction for misconduct, the modest correlation coefficient (r = −0.12) suggests that only ∼1% of the variance in time-to-retraction can be explained by higher scrutiny. Therefore, scrutiny is significantly related to the risk of retraction for misconduct but does not appear to be a major factor. We repeated this analysis after deleting all authors with more than a single retraction (Table S1) and found that the results were essentially the same; detection of error was significantly more likely in journals with a high IF, but this explained only ∼1% of the variance in time-to-retraction.

To determine whether the use of plagiarism-detection software may have increased the detection of plagiarism in published articles, we specifically evaluated the time required to retract articles containing plagiarized text. The time to retract an article for plagiarism is substantially shorter than the time to retract an article for misconduct (Table 1), which suggests that plagiarism-detection software is not responsible for an increase in the retraction of older articles. The ability to test this hypothesis rigorously is limited, as plagiarism is a relatively recent rationale for retraction. The first article retracted for plagiarism was published in 1979, and only 41 articles were retracted for plagiarism prior to 2007. Although the mean time-to-retraction for plagiarism was 26.0 months, retraction for plagiarism can occur after an extraordinarily long interval; one article was retracted for plagiarism in 2009 after 208 months in the literature. Because retraction for plagiarism is an infrequent event (192 retractions for plagiarism in total, or 9.4% of all retractions), these results cannot account for much of the recent spike in retractions. We note also that the first article retracted for scientific error was published in 1979 and the first article retracted for duplicate publication was published in 1990. This shows that articles are now retracted for reasons not cited in the past.

**Figure 2:**
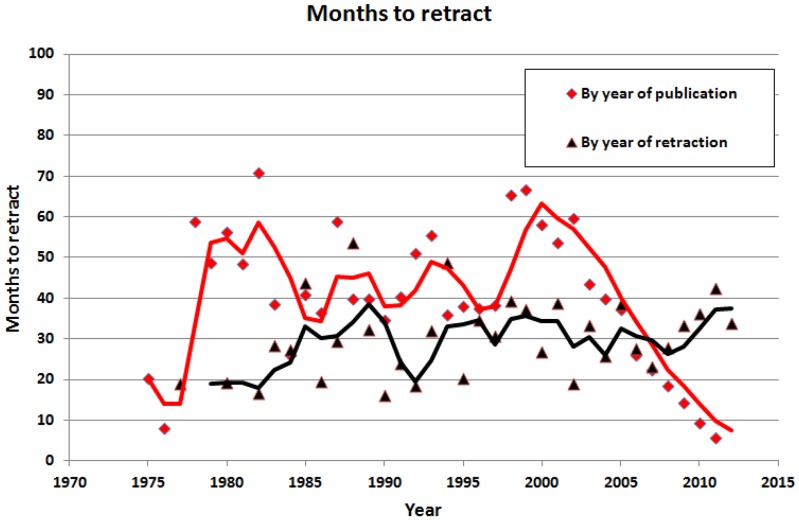
Months to retract by year of publication and by year of retraction. The fitted lines are 3-year moving averages of the plotted points. Apparent declines in recent years must be interpreted with caution as additional papers may be retracted in the future, thereby reversing this decline.

The recent increase in retractions is consistent with two hypotheses: (1) infractions have become more common or (2) infractions are more quickly detected. If infractions are now more common, this would not be expected to affect the time-to-retraction when data are evaluated by year of retraction. If infractions are now detected more quickly, then the time-to-retraction should decrease when evaluated as a function of year of publication. To test these hypotheses, we evaluated time-to-retraction as a function of both year of publication and year of retraction. Since 2000, there has been a progressive decline in the time-to-retraction, when analyzed by year of publication (Fig. 2), consistent with the hypothesis that manuscripts warranting retraction are now detected more quickly. This change has affected fraud and error to approximately the same degree (data not shown), suggesting that there may now be a lower barrier to retraction overall.

Retraction may be occurring more quickly now than in the past. Among 714 retracted articles published between 1973 and 2002, retraction required an average of 49.82 (±44.78 SD) months. Among 1,333 retracted articles published after 2002, retraction required 23.82 (±22.17 SD) months (p<0.0001). We note that misconduct takes longer to retract than any other type of offense (Table 1), which may mean that misconduct takes longer to prove. However, an important limitation of this analysis is that many articles published in recent years may yet be retracted in future years, which could eventually increase the time-to-retraction for recent articles.

To test the hypothesis that authors with multiple retractions have become more common in recent years and have had a major impact on the rise in retractions, we sought to characterize authors with >5 retractions. Among 409 retracted articles written by authors with >5 retractions since 1973, 337 (82.4%) were published in the last 20 years (since 1992), and 245 (59.9%) were published within the last decade. The time-to-retraction for 409 articles by authors with >5 retractions was 52.36 months (±37.95 SD), significantly (p<0.0001) longer than the time-to-retraction for 1,638 articles by authors with ≤5 retractions (28.03 months ±31.03 SD).

Authors with multiple retractions have had a considerable impact, both on the total number of retractions and on time-to-retraction. In 2011, 374 articles were retracted; of these, 137 articles (36.6%) were written by authors with >5 retractions. Articles retracted after a long interval (≥60 months after publication) make up 17.9% of all retracted articles; approximately two-thirds (65.7%) of such articles were retracted due to fraud or suspected fraud, a rate of fraud higher than in the overall sample [8]. Among fraudulent articles retracted ≥60 months after publication, only 10.4% (25/241) were written by authors with a single retraction.

**Figure 3:**
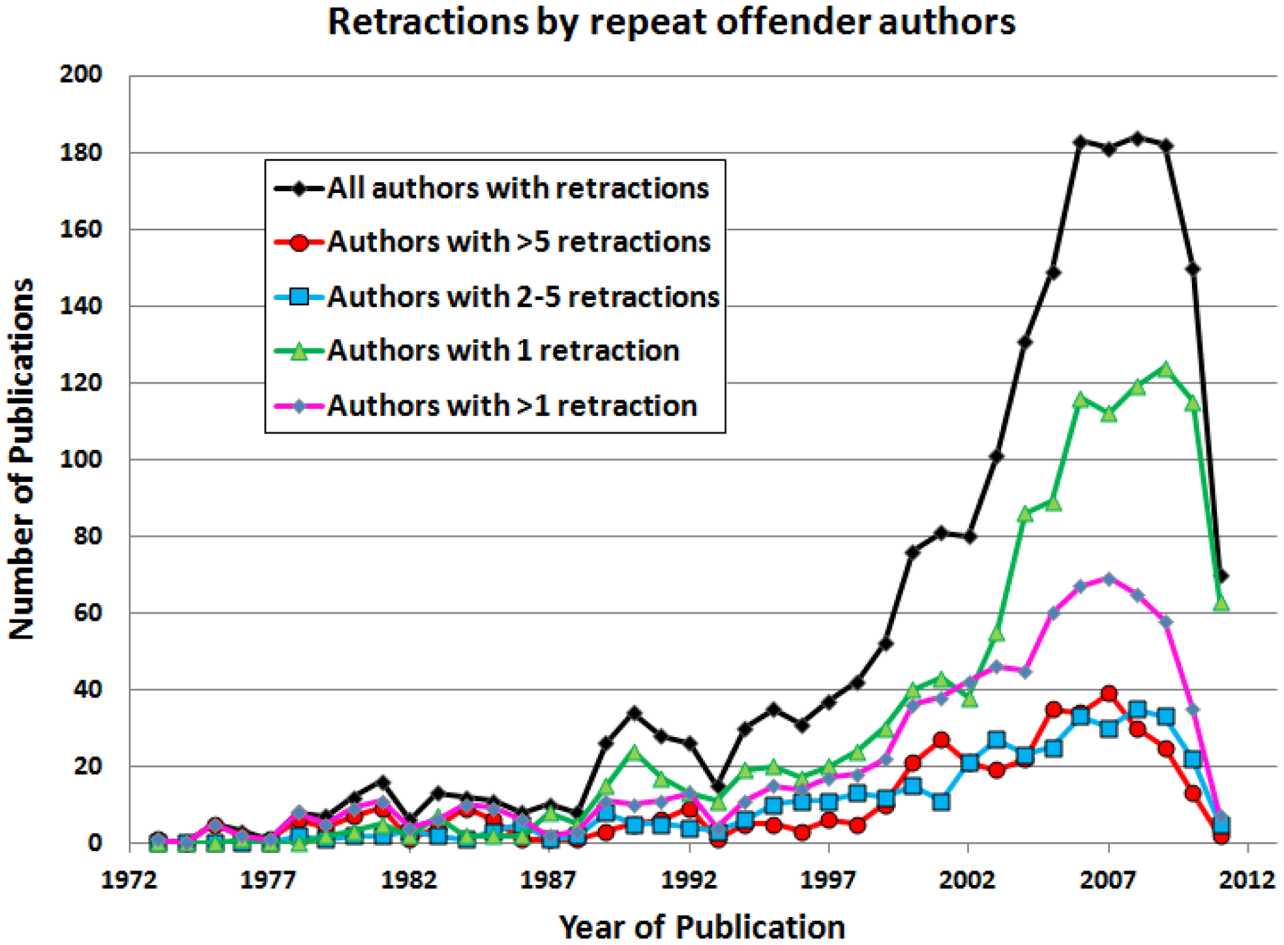
Retractions by authors with varying numbers of retractions, plotted by year. Apparent declines in recent years must be interpreted with caution as additional papers may be retracted in the future, thereby reversing this decline.

**Figure 4:**
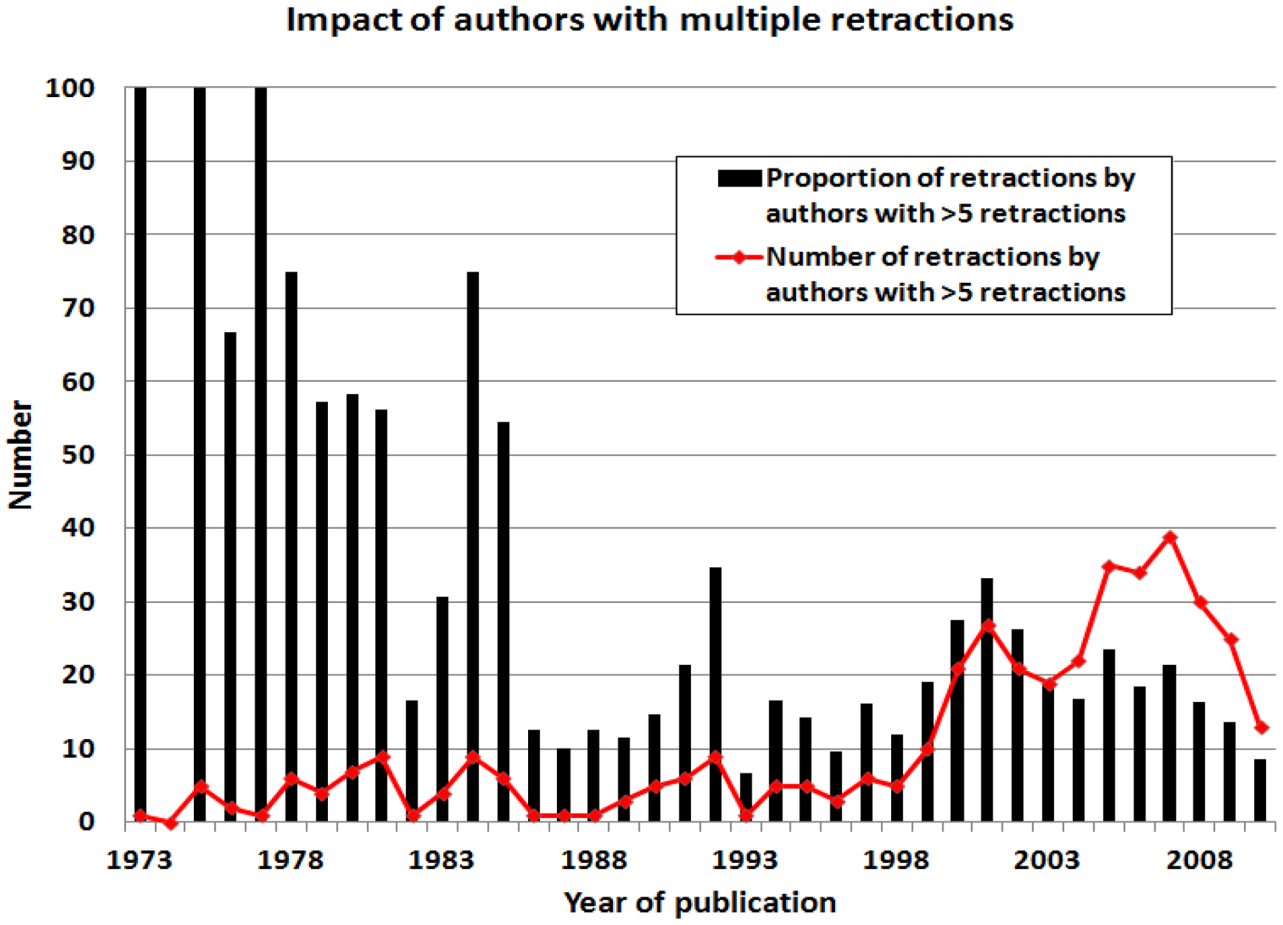
Impact of authors with >5 retracted articles, plotted by year. Apparent declines in recent years must be interpreted with caution as additional papers may be retracted in the future, thereby reversing this decline.

When authors with multiple retractions are evaluated by year of retraction, it becomes clear that authors with a single retraction have had more impact on the recent surge in retractions than have authors with multiple retractions (Fig. 3). Figure 3 shows the number of publications by year; authors with >5 retractions published 34.5% of all retracted papers from 1972 to 1992, whereas these authors published 17.8% of all retracted papers from 1993 to 2012. The number of retracted papers by authors with >5 retractions increased from 81 to 323 in these two time periods, but the proportional contribution has been smaller (χ^2^ = 35.45; p<0.001) over the past 40 years (Fig. 4). In contrast, from 1972–1992, 46.0% of retracted papers were written by authors with a single retraction; from 1993 to 2012, 63.1% of retracted papers were written by single-retraction authors (χ^2^ = 25.66; p<0.001).

Papers by authors with one retraction are fundamentally different from papers by authors that have more than one retraction (Table S2). Papers by authors with multiple retractions are withdrawn after a longer period of time (P<0.001) and are significantly more likely to involve misconduct (p<0.001). In contrast, papers by authors with a single retraction are significantly more likely to be retracted for plagiarism, error, duplicate publication, other causes, or unknown causes (all p<0.01).

Authors with multiple retractions have had a major impact on time-to-retraction. It is unusual for articles to be retracted for fraud >60 months after publication unless the article is part of a serial-fraud incident. Boldt and his associates [23] were responsible for 69 retractions for fraud in 2011; among his retracted articles, average time to retraction was 79.7 months (range; 6–149 months) and 46 articles (66.7%) were retracted ≥60 months after publication. Similarly, Mori and his associates had 28 retractions for fraud in 2011 [24]; the average time-to-retraction was 56.4 months and 11 articles (39.3%) were retracted ≥60 months after publication. Together Boldt and Mori were responsible for 25.9% of all articles retracted in 2011. Of 409 retracted articles written by authors with >5 retractions, 59.9% were published within the last decade, and such articles account for 20% of all retractions overall.

**Figure 5:**
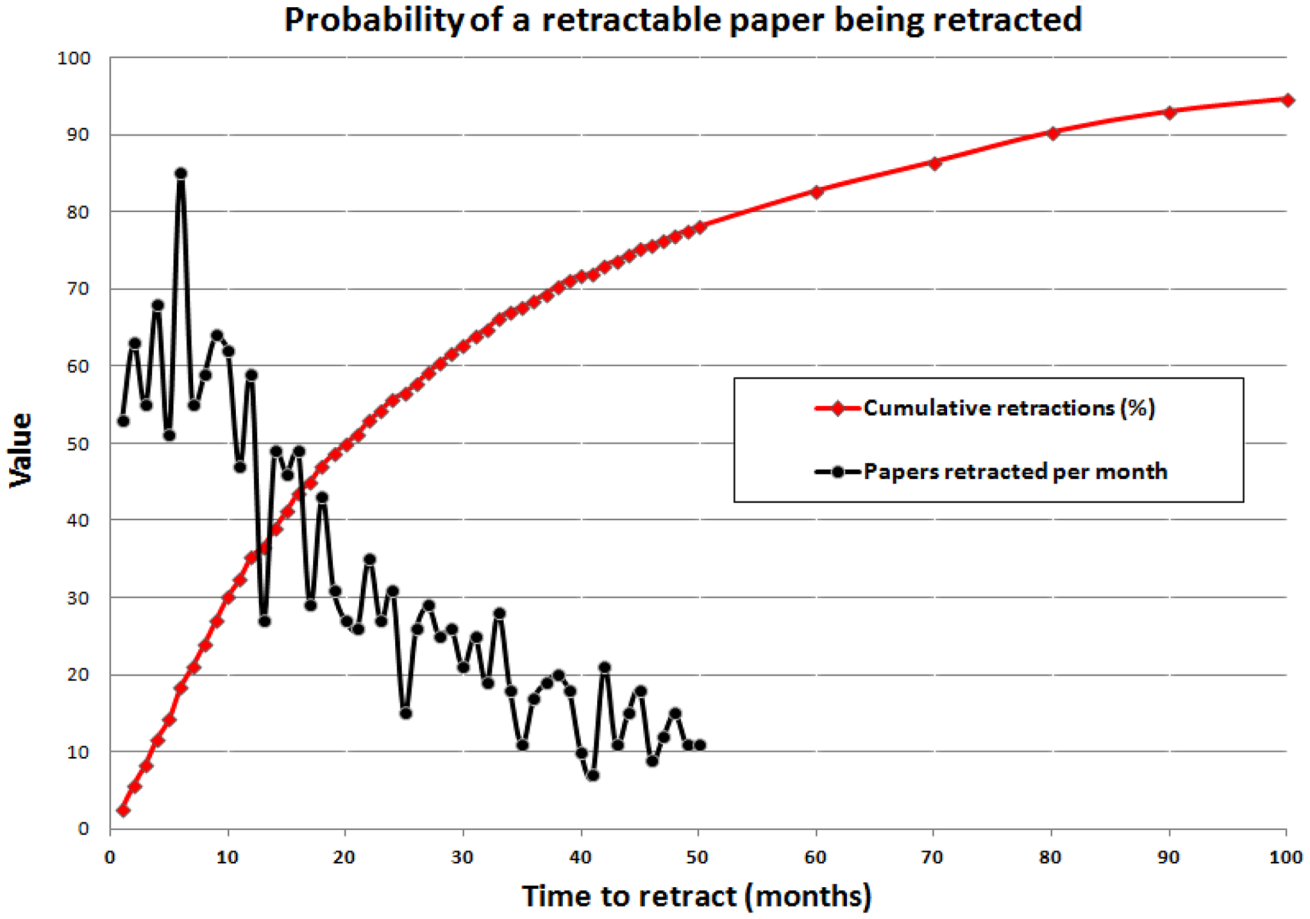
Cumulative probability of a retractable paper being retracted, together with the number of papers retracted per month.

The hypothesis that journals retract articles more rapidly now than in the past is weakened by the consideration that there has been little time to recognize flawed articles published recently. To test if retraction-worthy articles published recently have simply not been recognized yet, a model was developed to predict future retractions. This model assumes that all retractable articles will be recognized and retracted within 200 months (16.7 years) of publication (Fig. 5). The risk of retraction each year was determined from only two parameters: (1) the number of articles retracted by year *Y* after publication, and (2) the proportion of all retractable articles expected to be recognized and retracted by year *Y*. The proportion of retractable articles retracted over time is shown (Fig. 5). Articles are more likely to be retracted 6 months after publication than at any month before that, and the likelihood of retraction falls progressively thereafter. Cumulative probability that a retractable article will be retracted within 1 year is 35.2%, and the probability within 5 years is 82.6%. Approximately half of the articles that require retraction are retracted within 20 months of publication.

Using the cumulative probability of retraction (Fig. 5), the corrected number of retractions χ can be calculated as follows:

Where ρ = Number of articles already retracted by year *Y* after publication.

π = Cumulative probability of retraction by year *Y.*

**Figure 6:**
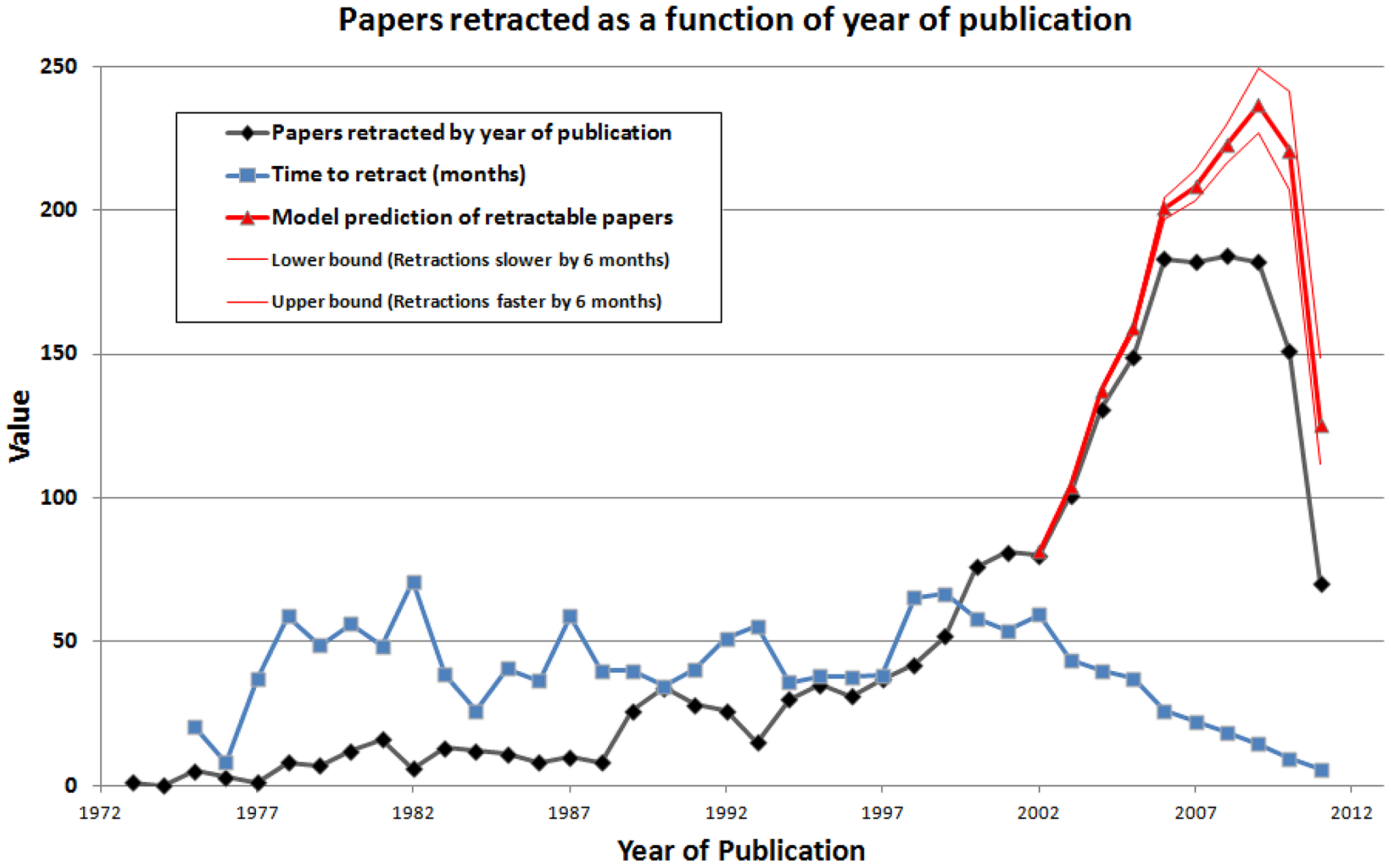
Articles retracted as a function of year of publication, shown with model predictions of the number of papers likely to be retracted. Apparent declines in recent years must be interpreted with caution as additional papers may be retracted in the future, thereby reversing this decline.

For example, of the articles published in 2010, 151 articles had been retracted when this database was constructed. To calculate, in 2013, the number of articles published in 2010 that are likely to be retracted eventually, only one additional piece of information is required; the cumulative probability of retraction after 3 years, which is 0.6841. If all retractable articles published in 2010 were to be retracted, the model predicts an eventual total of 221 ( = 151/0.6841) retractions (Fig. 6). An upper bound for this prediction was estimated by assuming that retractions are now occurring six months faster than the historical average (Fig. 6), while a lower bound for the prediction was estimated by assuming that retractions are now happening six months slower than the historical average. The model suggests that the rising rate of retractions may have stabilized. However, an important caveat is that prior patterns of retractions may not necessarily be predictive of future events.

## Discussion

A substantial increase in the rate of retracted scientific articles has been observed [8]. The present study analyzed several hypotheses that might account for this increase, with an emphasis on the time interval between publication and retraction. Evidence supports contributions from the following factors:

- The rate of publication has increased, with a concomitant increase in the rate of retraction (Fig. 1).
  - Editors are retracting articles significantly faster now than in the past (Fig. 2).
  - The reasons for retraction have expanded to include plagiarism and duplicate publication.
- Journals are reaching further back in time to retract flawed work.
- There has been an increase in the number and proportion of retractions by authors with a single retraction (Fig. 3).
- Discovery of fraud by an author prompts reevaluation of an author’s entire body of work.
- Greater scrutiny of high-profile publications has had a modest impact on retractions (Table 1).

The recent spike in retractions thus appears to be a consequence of changes both in institutional policy and in the behavior of individual authors.

The phenomenon of retraction itself appears to be a relatively recent phenomenon. Although the PubMed database lists biomedical research publications since 1966, along with selected articles published prior to that time, the earliest publication indexed as a retracted article in PubMed was published in 1973 and retracted in 1977 [25]. Yet it is clear that scientific misconduct resulted in fraudulent publications before 1977 [26]–[28]. Similarly, the first articles retracted for error or plagiarism were published in 1979, and the first article retracted for duplicate publication was published in 1990. Retraction is more widely recognized as a remedy for a flawed publication in the modern era, and the reasons for retraction have expanded over time.

Authors responsible for multiple retracted articles have received a great deal of attention [8], [23], [29]–[33], and our results show that they have had a considerable impact on the literature. Prior to the most recent decade, authors with >5 retractions (Fig. 4) were a few highly prolific scientists, including Robert Gullis, who misrepresented hypotheses as experimental results in 8 articles [25], John Darsee who authored 13 articles later retracted for data fabrication [34], [35], and Robert Slutsky, who had 17 articles retracted for fraud [29]. Recognition of serial misconduct has increased in recent years, although retractions by authors with only one retraction are more common (Fig. 3) and proportionally more important (Fig. 4). Nevertheless, research groups led by Joachim Boldt and Naoki Mori were responsible for 25.9% of all articles retracted in 2011, suggesting that these individual authors have had a grossly disproportionate impact on retractions from the literature.

Once a fraudulent article is detected, institutional investigation of the author’s work frequently uncovers additional instances of fraud [35]. However, the process of scrubbing the literature to remove the influence of a serial offender can be very lengthy. For example, a problem was noted in 2000 with the research output of the Japanese anesthesiologist Yoshitaka Fujii, whose data showed an abnormal absence of variability in the side effects of medication [36]. More recent follow-up suggests that Fujii’s publications, which still had not been retracted at the time this database was assembled, may involve extensive fraud [37]. Examination of 168 randomized clinical trials (RCTs) published by Fujii demonstrates that these trials contain extremely aberrant data distributions. The distribution of variables in individual RCTs were inconsistent with expected values in 96 of 134 human studies by Fujii [37]. The age distribution of subjects in one large study showed a highly non-random distribution, though no exclusion criteria were noted that could explain this distribution. The likelihood of obtaining this distribution by chance alone was *P*<10^−33^. Subsequent to when this database was assembled, the *Canadian Journal of Anesthesia* retracted 17 fraudulent papers by Fujii which had been published in that journal and indicated that a further 17 articles were “indeterminate” for fraud [38]. It seems likely that many more articles by this author will be retracted in the future [37], though Fujii maintains his innocence [39]. It is noteworthy that it has taken more than a decade for the investigation of Fujii’s work to proceed from suspicion to retraction.

The work reported here has several limitations. Many articles published recently could be retracted in the future, which might alter the average time-to-retraction (Table 1). A change in time-to-retraction could alter the calculation of the cumulative probability of a retractable paper being retracted (Fig. 5). If there is a change in the cumulative probability of retraction, this would in turn alter the estimate of the number of articles likely to be retracted in the future (Fig. 6). A single author with a large number of retractions, such as Boldt or Fujii, could markedly change the conclusions that the data now suggest. Another limitation of our study is that it does not address flawed work that has not been retracted.

Data fabrication and falsification are not new phenomena in science. Gregor Mendel, the father of genetics, may have modified or selectively used data to support his conclusions [40] and statistical analysis suggests that Mendel’s “data… [are] biased strongly in the direction of agreement with expectation…. This bias seems to pervade the whole data [set]” [26]. However, there now appear to be lower barriers to retraction as a remedy to correct the scientific literature. Our results (Fig. 5) suggest that the overall rate of retraction may decrease in the future as editors continue to process a glut of articles requiring retraction. Better understanding of the underlying causes for retractions can potentially inform efforts to change the culture of science [41] and to stem a loss of trust in science among the lay public [42], [43].

## Supporting Information

Table S1 **Correlations among journal impact factor (IF) and time-to-retraction expressed in months for different infractions, after deleting all authors with more than one retraction.** The correlation coefficient r is tested for significance with the R statistic, which has a t-distribution.
(DOCX)

Table S2 **Comparison of papers by authors with one retraction to papers by authors with multiple retractions.** Differences in months to retract and average journal impact factor (IF) were tested with a t-test. Differences in type of infraction were tested by χ^2^ analysis, by collapsing all differences into a 2×2 contingency table. Asterisks indicate values which are higher than expected by χ^2^ analysis.
(DOCX)

Database S1 **Excel file listing all retracted articles analyzed in this paper.** The file includes first author, article title, journal of publication, year of publication, year of retraction, months to retract, and the PubMed Identifier (PMID).
(XLSX)
