# Correction: Why Has the Number of Scientific Retractions Increased?

**DOI:** 10.1371/annotation/0d28db18-e117-4804-b1bc-e2da285103ac

**Published:** 2013-07-19

**Authors:** R. Grant Steen, Arturo Casadevall, Ferric C. Fang

In the Results section of the article, just before the last paragraph of the section, there was an equation omitted.

The full, relevant, section with the equation included is:

Using the cumulative probability of retraction (Fig. 5), the corrected number of retractions χ can be calculated as follows:

χ = ρ / π

Where ρ = Number of articles already retracted by year Y after publication.

π = Cumulative probability of retraction by year Y.

