# Supplementary material for: Why Has the Number of Scientific Retractions Increased?
Source: PLoS One. 2013 Jul 8;8(7):e68397. doi: 10.1371/journal.pone.0068397 (PMC3704583; doi:10.1371/journal.pone.0068397)
Supplement: Table S1 — Correlations among journal impact factor (IF) and time-to-retraction expressed in months for different infractions, after deleting all authors with more than one retraction. The correlation coefficient r is tested for significance with the R statistic, which has a t-distribution. (DOCX) [file pone.0068397.s001.docx]

**Steen et al-Supplementary material-PONE-D-12-27236R1**

**Table S1.** Correlations among journal impact factor (IF) and time-to-retraction expressed in months for different infractions, after deleting all authors with more than one retraction. The correlation coefficient r is tested for significance with the R statistic, which has a t-distribution.

|  |  | **Journal IF** |  | **Months to retract** | | **Correlation r** |  |  |
| --- | --- | --- | --- | --- | --- | --- | --- | --- |
|  | **Sample n** | **Mean** | **SD** | **Mean** | **SD** | **IF x Months** | **R=** | **P <** |
| **Misconduct + Poss. misconduct** | 326 | 9.09 | 10.94 | 30.79 | 30.79 | -0.020 | -0.36 | NS |
| **Misconduct** | 205 | 9.94 | 11.32 | 34.93 | 32.24 | -0.069 | -0.99 | NS |
| **Possible misconduct** | 121 | 7.65 | 10.14 | 23.77 | 26.87 | 0.032 | 0.35 | NS |
| **Plagiarism** | 145 | 2.75 | 2.50 | 21.92 | 29.68 | -0.097 | -1.17 | NS |
| **Error** | 357 | 11.13 | 11.74 | 23.41 | 20.85 | 0.104 | 1.97 | 0.03 |
| **Duplicate publication** | 200 | 3.90 | 6.56 | 22.16 | 24.99 | -0.107 | 1.51 | NS |
| **All single retractions** | 1250 | 7.03 | 9.66 | 24.54 | 27.23 | 0.035 | 1.24 | NS |
