# Supplementary material for: Why Has the Number of Scientific Retractions Increased?
Source: PLoS One. 2013 Jul 8;8(7):e68397. doi: 10.1371/journal.pone.0068397 (PMC3704583; doi:10.1371/journal.pone.0068397)
Supplement: Table S2 — Comparison of papers by authors with one retraction to papers by authors with multiple retractions. Differences in months to retract and average journal impact factor (IF) were tested with a t-test. Differences in type of infraction were tested by χ2 analysis, by collapsing all differences into a 2×2 contingency table. Asterisks indicate values which are higher than expected by χ2 analysis. (DOCX) [file pone.0068397.s002.docx]

**Steen et al-Supplementary material-PONE-D-12-27236R1**

**Table S2.** Comparison of papers by authors with one retraction to papers by authors with multiple retractions. Differences in months to retract and average journal impact factor (IF) were tested with a t-test. Differences in type of infraction were tested by χ^2^ analysis, by collapsing all differences into a 2 X 2 contingency table. Asterisks indicate values which are higher than expected by χ^2^ analysis.

|  | **Papers by authors** | **Papers by authors** |  |  |
| --- | --- | --- | --- | --- |
|  | **with 1 retraction** | **with >1 retraction** | **T-test** | **χ^2^** |
|  | (n = 1250) (%) | (n = 797) (%) | **significance** | **significance** |
| Months to retract | 24.54 | 46.04 | < 0.001 | / |
| Misconduct | 205 (16.4%) | 492 (61.7%) | / | < 0.0001 |
| Possible misconduct | 121 (9.7%) | 71 (8.9%) | / | NS |
| Plagiarism | 145 (11.6%) | 55 (6.9%) | / | < 0.01 |
| Error | 357 (28.6%) | 80 (10.0%) | / | < 0.001 |
| Duplicate | 200 (16.0%) | 90 (11.3%) | / | < 0.01 |
| Other | 102 (8.2%) | 6 (0.8%) | / | < 0.001 |
| Unknown | 154 (12.3%) | 28 (3.5%) | / | < 0.001 |
| Average journal IF | 7.03 | 7.73 | NS | / |
|  |  |  |  |  |
